# Adoption and impact of improved groundnut varieties on household food security in Nigeria

**DOI:** 10.1016/j.jafr.2023.100817

**Published:** 2023-12

**Authors:** Mequanint B. Melesse, Philip Miriti, Geoffrey Muricho, Chris O. Ojiewo, Victor Afari-Sefa

**Affiliations:** a International Crops Research Institute for the Semi-Arid Tropics (ICRISAT), Kenya; b International Maize and Wheat Improvement Center (CIMMYT), Kenya; c International Crops Research Institute for the Semi-Arid Tropics, India

**Keywords:** Improved groundnut variety, Groundnut consumption, Food security, Endogenous switching regression, Nigeria

## Abstract

Improved agricultural technologies are promoted as cost-effective and sustainable ways of improving rural households' food security and reducing poverty in developing countries. This study evaluates the relationship between improved groundnut varieties (IGVs) and household food security using detailed household and plot level data from a sample of over 1300 farm households in Northern Nigeria. Endogenous switching regression models are employed to control for potential endogeneity biases. Results show that about 30 % of groundnut plots are planted with improved varieties, and the adoption of IGVs significantly increases the likelihood of household per capita groundnut consumption by about 13 % and reduces the probability of households' vulnerability to food (access) insecurity by 22 %. Counterfactual analyses show that non-adopting households could have enjoyed comparable benefits had they adopted IGVs. These results suggest that development interventions aimed at improving the diffusion and impacts of IGVs in Nigeria need to target farmers’ access to information about the technologies while developing groundnut seed systems to make quality seeds readily available to smallholder farmers at affordable prices.

## Highlights

• Many improved groundnut varieties have been developed over the years in Nigeria.
• We investigate adoption and impact of improved groundnut varieties on household groundnut consumption and food security.
• Improved varieties adopted on 30 % of planted groundnut plots.
• Adoption of improved groundnut varieties significantly improves household groundnut consumption and food security.

## Graphical abstract

**Figure:**
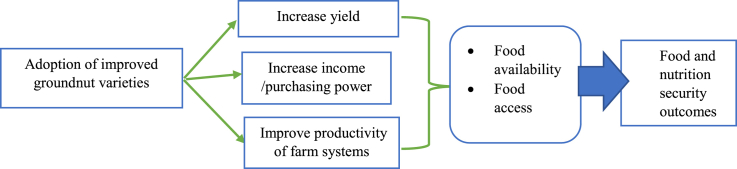

## 1 Introduction

Malnutrition in its various forms remains one of the most pressing global challenges with huge social and economic costs [1,2]. Addressing this challenge requires increasing agriculture productivity and production substantially to meet the demand for affordable and nutritious food for the ever growing rural and urban population in the developing world [3,4]. Improved varieties of food crops have long been recognized as cost-effective and more sustainable approaches to increasing food production and improving rural incomes and livelihoods [5,6]. Evidently, many empirical studies have shown that adoption of improved agricultural technologies have significant impact on increasing productivity, improving food security and reducing poverty in Africa, for example [[7], [8], [9], [10]].

In this study, we investigate the relationship between adoption of improved groundnut varieties (IGVs) and rural household food security in Nigeria, a country with high levels of poverty and malnutrition. Groundnut (*Arachis hypogaea*) is an important crop in smallholder farming systems, contributing to food security and poverty reduction [11,12]. Groundnut can contribute to household food security in several ways. First, groundnut is an important part of diets throughout Africa, supplying protein, healthy fats, vitamins and micronutrients to people who often rely on starchy grains, roots and tubers [13]. Notably, it is an important source of affordable protein for the rural and urban poor who cannot afford animal products and can contribute to improving household nutrition and health through consumption of plant-based protein. Second, groundnut is a cash crop and its production accounts for up to 50 % of cash income for households in Africa [12], increasing their purchasing power to diversify diets through market purchase. Third, groundnut is an essential component of cropping systems in smallholder agriculture, as a sole crop, relay, or inter cropping with other crops, like millets and sorghum [12], improving the productivity of the overall farm system of households. Groundnut production improves soil fertility in drylands of sub-Saharan Africa (SSA) due to its high potential to fix nitrogen. Its haulms and residues also serve as high-quality livestock feed. Groundnut uniquely supports women's livelihoods and empowerment, because it is grown predominantly by women for subsistence [12,13].

Given its importance to rural livelihoods and economic growth, considerable research has been devoted to the development and dissemination of IGVs in Nigeria. Over the past 50 years, international and national research institutions, such as the Institute for Agricultural Research (IAR) of Nigeria, have developed and promoted IGVs that are high yielding, drought tolerance and resistant to multiple diseases. The Tropical Legumes (TL) project, for example, has supported the release and dissemination of several IGVs in Nigeria. Funded by the Bill & Melinda Gates Foundation (BMGF), TL was implemented in three phases from 2007 to 2019 in collaboration with international agricultural research institutes and national agricultural research systems (NARS). The project has played a significant role in creating awareness about IGVs and improving groundnut seed systems [14]. Taken together, over 30 high yielding and stress-tolerant groundnut varieties have been released in Nigeria to date [15].

Adoption of IGVs can improve smallholder household food security through several impact pathways, including increased yield, better income, and lower downside risk of smallholder farming systems in changing market conditions. However, despite increasing efforts on genetic improvement of groundnut, there is limited empirical evidence on impacts of IGVs on household food security in Nigeria, for example [10]. Such evidence is needed to justify continued investments in agricultural research for the development of market-led and climate-resilient groundnut varieties and to understand whether these technologies ultimately affect household food security. Previous studies on adoption and impacts of IGVs in Nigeria mainly focused on understanding the patterns and drivers of adoption and yield evaluations, for example [11,13,14,16]. Additionally, these studies are highly localized and covered few states that are not representative of groundnut growing areas of the country.

Our analysis relies on comprehensive household and plot level data from a sample of more than 1300 groundnut-growing households in Nigeria. The empirical strategy is based on the endogenous switching regression (ESR) approach, which accounts for potential endogeneity threats due to both unobserved heterogeneity and observed covariates [17]. This is a relevant improvement over the propensity score matching methods employed in some of the above-mentioned studies, as these approaches control only for observed covariates [18,19]. Our results show that about 30 % of groundnut plots were planted with improved varieties, and adoption of IGVs significantly increased household food security, in terms of more per capita groundnut consumption and improved food security outcomes. Overall, the findings reveal that agricultural research that leads to the development and dissemination of improved varieties can be a key driver of improving rural food security.

The rest of the paper is organized as follows. Section 2 describes the data and empirical strategy of the paper. Section 3 presents and discusses the results, while Section 4 concludes.

## 2 Data and research methodology

### 2.1 Data and descriptive statistics

#### 2.1.1 Sampling design

This study uses survey data collected in 2017 during the third-phase of the TL project in Northern Nigeria. The survey covered five states: Bauchi, Kebbi, Katsina, Kano and Jigawa. These states are located in the Guinea Savanna zone of Nigeria and represent the main agroecological zone suitable for groundnut production in the country [20]. Together, they account for about 65 % of the groundnut production in Nigeria. A multistage sampling was employed to select study households. First, three Local Government Authorities (LGAs) were randomly selected from each state. Second, two TL treated villages along with one non-TL village were randomly sampled to obtain 3 villages from every sampled LGA. Third, a sampling frame of groundnut growing households was developed with the help of agricultural extension agents and local officials in the selected villages. A probability proportionate to size (PPS) sampling was used to randomly select households from each village. Depending on the farming population in each village, the range limit was 10–35 households per village. This procedure produced a total sample size of 1470 groundnut producers. However, our analysis is based on 1311 households due to missing significant adoption and outcome data.^1^ These households operated on 1694 groundnut plots.

The survey collected valuable information on characteristics of the sampled households, including detailed demographics, income, land and livestock holdings, production systems, and household food security outcomes. In addition to household data, the survey solicited community level information on access to markets, credit and agricultural extension. Table 1 presents description of the sample by adoption status (1 = adopters and 0 = non-adopters).

**Table 1:** Summary statistics of variables by adoption of the IGVs.

Variable	Descriptions	Pooled	Adopters	Non-adopters	Difference (two-sample t-tests)
Adoption of IGVs	Household planted improved groundnut varieties (IGVs) in the 2016 cropping season (1 or 0)	0.30 (0.46)	100	0	
Per capita groundnut consumption	Household per capita groundnut consumption in kilograms (kg)	10.79 (23.63)	13.12 (26.54)	9.79 (15.01)	−3.34*
HFIAS score	Household food insecurity (access) scale score ranging from 0 to 27, indicating least vulnerable to most vulnerable to food insecurity conditions	2.34 (4.10)	1.69 (3.00)	2.62 (4.46)	0.936***
Food secure	Household experienced none of the food insecurity conditions (1 or 0)	0.56 (0.50)	0.59 (0.49)	0.55 (0.50)	−0.04*
Male household head	Household head is male (1 or 0)	0.92 (0.27)	0.90 (0.31)	0.93 (0.25)	0.036**
Age of head	Age of the household head in years	45.83 (11.91)	46.04 (10.97)	45.73 (12.29)	−0.303
Education of head	Household head completed junior secondary school (1 or 0)	0.16 (0.37)	0.15 (0.36)	0.17 (0.37)	0.013
Household size	Number of household members in the family	6.89 (3.49)	6.75 (3.58)	6.95 (3.45)	0.198
Livestock TLU	Livestock owned by a household in tropical livestock units (TLU)	1.87 (3.94)	1.94 (3.11)	1.84 (4.25)	−0.095
Cultivated land	Land under groundnut by households in hectares (ha)	2.26 (15.24)	2.49 (21.29)	2.17 (11.75)	−0.321
Extension access	Household was visited by extension agent (1 or 0)	0.61 (0.49)	0.80 (0.40)	0.53 (0.50)	−0.271***
Crop rotation	Farmer practiced crop rotation on plot (1 or 0)	0.44 (0.50)	0.42 (0.49)	0.45 (0.50)	0.032
Household per capita income	Household per capita income (‘000)	73.70 (248.60)	105.53 (317.36)	60.07 (211.15)	−45.462***
Credit amount	Amount of credit (‘000)	7.56 (31.89)	9.34 (21.78)	6.79 (35.33)	−2.546
Tropical legumes village	Farmer was from a village where the Tropical Legumes project implemented (1 or 0)	0.68 (0.47)	0.87 (0.33)	0.59 (0.49)	−0.279***
Variety awareness	Farmer had knowledge about improved varieties from formal information sources (1 or 0)	0.36 (0.48)	0.81 (0.39)	0.17 (0.38)	−0.636***
**State dummies**					
Bauchi (Reference)	Respondent was from Bauchi state (1 or 0)	0.20 (0.40)	0.15 (0.36)	0.22 (0.41)	0.061***
Jigwa	Respondent was from Jigwa state (1 or 0)	0.25 (0.43)	0.41 (0.49)	0.18 (0.38)	0.232***
Kano	Respondent was from Kano state (1 or 0)	0.18 (0.38)	0.10 (0.30)	0.22 (0.41)	0.119***
Kastina	Respondent was from Kastina state (1or 0)	0.18 (0.39)	0.24 (0.43)	0.16 (0.36)	−0.088***
Kebbi	Respondent was from Kebbi state (1 or 0)	0.19 (0.40)	0.10 (0.30)	0.24 (0.42)	0.139***
*Observations*		1694	508	1186	1694

Standard deviations in parentheses. *p < 0.1 **p < 0.05 ***p < 0.01. HFIAS – Household food insecurity access scale.

#### 2.1.2 Adoption of improved groundnut varieties (IGVs)

In this study, adoption is defined at a plot level in terms of whether or not an IGV was planted on the plot in the 2016 cropping season. Overall, the IGV adoption rate was about 30 % among groundnut plots (Table 1). Table A1 in the Appendix shows farmer reported adoption rates of some of the popular varieties. SAMNUT-24 is the most widely adopted IGV, covering 26 % of the plots.

Released in 2011, SAMNUT-24 is a popular variety because it has shorter maturity, is resistant to rosette disease, and has high oil content and good seed quality with a yield potential of 2 tons/ha [13]. Interestingly, other IGVs have not yet been adopted at scale. SAMNUT-26, which is highly rosette resistance, high yielding and early maturing variety, covered about 2 % of the plots. Kwankwaso (13 %), Yardakar (10 %), and Maiborgo (10 %) are widely grown local varieties of groundnut in Nigeria.

#### 2.1.3 Household food security outcomes

Our outcome variable is household food security, which was measured using different indicators. The first indicator is household groundnut consumption. Household groundnut consumption, which is considered in the context of this study as a proxy indicator for household welfare, refers to the per capita groundnut that was consumed by the household in kilograms. As IGVs are productivity-enhancing technologies, adopters are expected to realize more yields, which subsequently should lead to increased groundnut consumption. Adopters of IGVs had significantly more groundnut consumption compared to non-adopters. On average, the per capita groundnut consumption for adopters was about 3 kgs more than that of non-adopters. The variance for per capita groundnut is rather higher, suggesting the need to address the skewedness in its distribution.

Second, we used household food insecurity access scale (HFIAS), which is a more formal measure of household food security that is commonly used to measure household vulnerability to food insecurity [21]. HFIAS is an experiential measure of food security that identifies situations where households have food insecurity due to inter-and intra-household food distributions. It captures household behaviors regarding anxiety and uncertainty related to household insecure food access over the preceding four weeks (30 days) [21,22]. HFIAS involves a set of 9 questions with an ordinal response scale of 0–3. Respondents were first asked whether their answer to each question was yes or no. When the answer to this occurrence question was “yes”, frequency of occurrence was asked to determine whether the condition happened rarely (once or twice), sometimes (three to ten times) or often (more than ten times) over the past four weeks. Table A2 in the Appendix contains the relevant questions.

The HFIAS score is calculated for each household by summing the frequency-of-occurrence for each question, ranging from 0 to 27 where the higher score implies higher levels of vulnerability to food insecurity. However, one concern with using HFIAS is that it assumes that differences between scores are linear, and the ordinal categories are viewed as interval numerical values. In practice, this may not be the case as the intensity of food insecurity would not likely remain stable between score values. To mitigate this concern, we constructed a dummy (Food secure), taking the value of 1 if the household experienced none of the food insecurity conditions and 0 if the household experienced at least one of the food insecurity conditions. Table 1 shows the summary statistics for both measures. The average HFIAS is about 2.34, with adopters of IGVs experiencing less food insecurity. Similarly, 56 % of the households are food secure, again with adopters being more likely to be food secure or experience none of the underlying food insecurity conditions.

In summary, the statistics reveal that adopters of IGVs have higher groundnut consumption, are less likely to be vulnerable to food insecurity, and are more likely to be food secure than non-adopters. While suggestive, such simple comparisons can be misleading about impacts of IGVs, as the two groups might systematically differ in their observed and unobserved characteristics. This is particularly true since several covariates are significantly different between adopters and non-adopters (Table 1). In the subsequent sections, we account for these concerns by employing empirical strategies that control for differences in observed and unobserved characteristics and estimate causal effects.

#### 2.1.4 Explanatory variables

We draw on economic theory and the empirical literature on adoption and impact of agricultural technologies as well as cropping practices related to groundnuts to identify explanatory variables e.g., Refs. [7,23]. Table 1 provides descriptions of explanatory variables. About 92 % of the households are headed by males, with average age of about 46 years, with about 16 % of them having completed junior secondary school. The average household size is about 7 members. Household resource endowment is proxied by cultivated land and livestock ownership. Livestock is measured using tropical livestock unit (TLU), which is a common unit used to quantify various livestock species to a single value. The amount of credit and access to extension services are captured as community level determinants of adoption and impacts of IGVs, while crop rotation is an indicator of cropping practices.

Results show that adopters and non-adopters significantly differ in many characteristics. Adopters generally tend to have higher household income and access to extension services. Importantly, adopters of IGVs are more likely to come from TL project villages and to be aware of IGVs from formal information sources (i.e., extension agents, farmer groups and research organizations). About 87 % of the adopters and 59 % of non-adopters are from TL project villages, while about 64 % more adopters than non-adopters are aware of IGVs.

### 2.2 Research methodology

#### 2.2.1 Conceptual framework

As rational decision-makers, farmers would consider potential benefits of new technologies when making adoption decisions [24]. A farmer will adopt an IGV if the perceived economic benefits from the variety exceed its cost of adoption, subject to technology availability and resources and information constraints. Thus, the adoption decision can be modeled as constrained optimization using the random utility framework. Formally, let $U_{1i}$ denotes the utility from the adoption of IGV and $U_{0i}$ denotes the utility from non-adoption (i.e. the counterfactual of planting local groundnut variety). For adoption to happen, the expected utility ($E\left(U_{i}^{*}\right)$) from adoption of the technology should be positive, i.e. $E\left(U_{i}^{*}\right)=U_{1i}-U_{0i}>0$. However, the net expected utility from the adoption of an IGV is not observable. We represent it with a latent variable ($D_{ij}^{*}$), which itself is determined by a set of observable variables, $Z_{ij}$, and an independently and identically distributed error term ($u_{ij}$), such that:

(1) $$D_{ij}^{*}=\alpha_{ij}Z_{ij}+u_{ij},\text{with}\;D_{ij}=\left\{ \begin{array}{c} 1\;if\;D_{ij}^{*}>0\\ 0\;if\;D_{ij}^{*}\leq 0 \end{array}\right.$$

Equation (1) is estimated for every plot i of household j and represents the selection or adoption decision. $\alpha_{ij}$ represents a vector of parameters to be estimated. This implies that we can observe the actual outcomes (household groundnut consumption and food security) as a function of IGV adoption and observed and unobserved household and plot characteristics.

#### 2.2.2 Empirical strategy

Our goal is to estimate the impact of IGVs on household groundnut consumption and food security. When households are not randomly exposed to the technologies, the adoption decision could be potentially endogenous. In most cases, farmers either self-select into adoption or the technologies are targeted to a certain group of farmers [25]. For example, farmers may decide to adopt a new technology due to their innate managerial and technical abilities in understanding and using new agricultural technologies. We use the endogenous switching regression (ESR) model to address selection bias and endogeneity threats. The ESR model simultaneously estimates separate outcome equations for adopters and non-adopters along with adoption equation using the full information maximum likelihood (FIML) estimator [26]. While it assumes normality, like the instrumental variables (IV) approach, ESR is more efficient than IV techniques [17]. It has the advantage of simultaneously controlling for factors affecting the treatment and disentangling factors influencing outcomes. ESR models also control for structural differences between adopters and non-adopters regarding the outcome functions [25].

The estimation of ESR model proceeds in two stages. The first stage involves estimating a probit model of adoption, while the second stage estimates appropriate models for each of the outcome variables correcting the selection problem. As defined, $D_{ij}$ is the binary variable resulting from the utility maximization and represents the observed adoption status of a plot where $D_{ij}=1$ if the household reported to have planted an IGV on a plot and $D_{ij}=0$ otherwise. Conditional on the adoption decision, the outcome equations can be represented by switching regimes as follows:

(2a) $$\text{Regime}\;1\;\left(\text{Adopters}\right):y_{1i}=\beta_{1}X_{1i}+\epsilon_{1i}\;if\;D_{ij}=1$$

(2b) $$\text{Regime}\;2\;\left(\text{Non}-\text{adopters}\right):y_{0i}=\beta_{0}X_{0i}+\epsilon_{0i}\;if\;D_{ij}=0$$

where $y_{1i}$ and $y_{0i}$ represent the outcomes of household i for each regime (1 = for adopters and 0 = non-adopters); $X_{1i}$ and $X_{0i}$ are vectors of exogenous covariates; $\beta_{1}$ *and* $\beta_{0}$ are vectors of parameters to be estimated, and $\epsilon_{1i}$ and $\epsilon_{0i}$ are random error terms. The error terms of the selection and the regime equations ($u_{ij}$, $\epsilon_{1i}$ and $\epsilon_{0i}$) are assumed to have a trivariate normal distribution with a zero mean vector and covariance matrix [26]. Nonzero correlation between the error terms of the selection equation ($u_{ij}$) and the outcome equations ($\epsilon_{1i}$ and $\epsilon_{0i}$) provides evidence of existence of endogenous switching.

Although the non-linearity in the selection equation makes the simultaneous identification of the adoption and outcome equations possible, including exclusion restrictions based on valid instrument(s) in the adoption equation is recommended for a more robust identification [27]. As often, the challenge is obtaining credible instruments. Previous studies used instruments related to information sources, including extension, radio information, market information and distance to inputs [23,27,28]. Farmers would adopt an improved variety only if they have information or knowledge about the variety. Following this, we consider factors that leverage variety dissemination and farmers' information access as instruments for IGV adoption. Specifically, we employ farmer's awareness about IGVs from formal sources (i.e., extension agents, farmer groups and research organizations) and whether a farmer's village was included in the TL project communities as instruments. While villages might not be randomly assigned to the TL project, their assignment is less likely to be influenced by individual household behavior. With this consideration, our instruments can largely be justified because information on IGV should affect groundnut consumption and food security through its effect on adoption.

The validity of an IV strategy rests on two criteria: the relevance and exclusion restriction criteria [29]. While the relevance of instruments can easily be established, the exclusion restriction is more difficult to compellingly satisfy and prove. Formally, a falsification test can be used to assess the validity of instrumental variables [28]. The idea is that instruments should not affect the outcome variables among non-adopter households. The results for the relevance and falsification tests of the instruments are in the Appendix (Table A3). The instruments can be considered relevant and valid, since they are significantly correlated with the adoption of IGVs (p < 0.01), but not correlated with the outcomes for non-adopter households.

We also present further statistical tests for the appropriateness of the IV approach (Table A4, Appendix). The Wu-Hausman and Durbin-Wu-Hausman tests confirm presence of endogeneity concerns for per capita groundnut consumption and HFIAS, as exogeneity is rejected for both outcomes (p < 0.05). However, these tests indicate that the endogeneity concern is not a threat for the food secure outcome. The Kleibergen-Paap test rejects that the endogenous regressor is weakly identified (p < 0.05). Finally, the Hansen's J statistic and Anderson-Rubin Wald tests (i.e., over-identification restriction tests) fail to reject the null of zero correlation between instruments and the error term of the models, implying that our instruments are reasonable exogenous and valid.

We estimate different model specifications based on the distributions of the specific outcome variable. Essentially, the framework for estimation of the ESR model remains similar for continuous, binary, censored, or count outcomes; it involves estimation of the selection and outcome equations. As our treatment variable is defined at plot level, we report standard errors clustered at the household level to control for the fact that some households have multiple plots and these observations are not mutually independent.

The first outcome is household per capita groundnut consumption. This outcome variable is continuous; therefore, we estimate the ESR model in Stata using the *movestay* package. The outcome equations are represented by switching regimes which are conditional on the status of the households’ adoption of the IGVs. These are represented as:

(3a) $$\text{Regime}\;1:y_{i1}=\beta_{1}X_{1i}+\varepsilon_{1i}\;if\;A_{i}=1$$

(3b) $$\text{Regime}\;2:y_{i0}=\beta_{0}X_{0i}+\varepsilon_{0i}\;if\;A_{i}=0$$

where $X_{1i}$ and $X_{0i}$ are the vectors of the explanatory variables assumed to be weakly endogenous; $\beta_{1}$ and $\beta_{0}$ are the vectors of the parameter estimates, and $\varepsilon_{1i}$ and $\varepsilon_{0i}$ as the error terms. The model is estimated using a full information maximum likelihood [26,30].

Next is HFIAS score, which is a non-negative count data ranging from 0 to 27. We use an endogenous switching Poisson (ESPo) model to estimate the impact of IGV on household food insecurity conditions. We use the *teescount* after *escount* command in Stata version 15 software to estimate the treatment effects [31]. Lastly, the “food secure” outcome is binary. We employ an endogenous switching probit (ESPr) framework. The ESPr model is estimated in Stata using the *switch_probit* packages [26].

#### 2.2.3 Treatment effects

Once the various models are estimated, the next step is to generate treatment effects by computing the conditional expectations or expected outcomes for adopters and non-adopters of IGVs (Table 2). The average treatment effect on the treated (ATT) is computed as the difference between the average expected outcomes (observed) and the counterfactual for adopters. Similarly, the average treatment effect on the untreated (ATU) is computed as the difference between the outcomes that non-adopters would have obtained had they adopted IGVs (counterfactual) and the expected outcomes of non-adopters (observed).

**Table 2:** Conditional expectations, treatment effects, and heterogeneous effects.

Subsamples	Decision stage		Treatment effects^e^
	To adopt	Not to adopt	
**Adopters**	$E\left(y_{1i}|D_{ij}=1\right)$^a^	$E\left(y_{0i}|D_{ij}=1\right)$^b^	Average treatment effect on the treated (ATT)
**Non-adopters**	$E\left(y_{1i}|D_{ij}=0\right)$^c^	$E\left(y_{0i}|D_{ij}=0\right)$^d^	Average treatment effect on the untreated (ATU)
**Heterogeneous effects**	*BH*_*1*_	*BH*_*0*_	Transitional heterogeneity (TH)

Notes: ^a^Conditional expectation of outcomes for adopters under observed condition; ^b^Conditional expectation of outcomes for adopters under the counterfactual condition; ^c^Conditional expectation of outcomes for non-adopters under the counterfactual condition; ^d^Conditional expectation of outcomes for non-adopters under the observed condition; ^e^The corresponding treatment effects are differences between the estimates in the respective rows; ${BH}_{i}$ is the effect of base heterogeneity for adopters ($D_{ij}=1)$ and non-adopters ($D_{ij}=0$).

It might be possible for households that adopted IGVs to have better outcomes (per capita groundnut consumption or food security) than non-adopter households regardless of the adoption status because of unobserved characteristics, such as risk behavior and entrepreneurial skills. The conditional expectation equations can also be used to estimate such base heterogeneity effects due to potential unobserved characteristics [28]. Another important parameter is transitional heterogeneity (TH), which measures whether the effect of IGVs is larger or smaller among the adopter or non-adopter households, in the counterfactual case that they had adopted [28]. TH is measured as the difference between the ATT and ATU (i.e. TH=ATT−ATU). Alternatively, it can also be computed as the difference between the base heterogeneity effects of adopters and non-adopters (${BH}_{1}-{BH}_{0}$).

Like the ESR models for continuous outcome variables, the switching Poisson and probit models allow for the estimation of ATT and ATU for the respective outcomes. In addition, these models estimate the average treatment effect (ATE), which is the measure of the impact of adoption of IGVs for households randomly selected from the population of households with given characteristics. Similarly, like the ESR for continuous outcomes, treatment effects in these models may vary due to unobserved characteristics [32]. However, unlike linear ESR models, the estimation procedure for ESPo and ESPr models does not generate heterogeneity effects. Instead, we account for unobserved heterogeneity effects by estimating marginal treatment effects (MTE) that control for the effect of IGVs on the outcomes for households that are motivated to change their outcomes because of the presence of IGVs [32].

## 3 Empirical results and discussion

### 3.1 Determinants of adoption of improved groundnut varieties

We first discuss the results of the selection probit model of the endogenous switching regressions and the corresponding marginal effects (Table 3). The results show that many factors are significant determinants of adoption of IGVs. The probability to adopt IGVs decreases with age of the household head, suggesting that younger households are more likely to try new agricultural technologies. Female-headed households are more likely to adopt IGVs as compared to those headed by men. This can partly be explained by the traditional culture of Nigerian farming households where groundnut production is considered as women's activity. While men and women cultivate and manage groundnut production in the country, the production of groundnuts relies heavily on women as the primary source of labor [11,20].

**Table 3:** Endogenous switching regression estimates of determinants of adoption of IGVs.

Variables	Coefficient	Std. error	Marginal effects	Std. error
Age of head	−0.355*	0.182	−0.077*	0.039
Male household head	−0.277**	0.136	−0.060**	0.029
Education of head	0.133	0.123	0.029	0.027
Household size	0.089	0.074	0.019	0.016
Livestock TLU	0.086	0.056	0.019	0.012
Cultivated land (ha)	−0.029	0.023	−0.006	0.005
Extension access	0.327***	0.106	0.071***	0.023
Crop rotation	−0.180*	0.093	−0.039*	0.020
Log household per capita income	0.0003**	0.0001	0.0001**	0.00003
Log credit amount	−0.126**	0.049	−0.027***	0.011
Tropical legumes village	0.303**	0.120	0.066**	0.026
Variety awareness	1.287***	0.087	0.279***	0.016
**State dummies**				
Bauchi (Reference)				
Kebbi	−0.529***	0.173	−0.115***	0.038
Kastina	0.290*	0.155	0.063*	0.033
Kano	−0.371**	0.175	−0.081**	0.038
Jigwa	−0.133	0.159	−0.029	0.035
Constant	−0.200	0.643		
Observations	1694		1694	

Note: APE – average partial effects estimated after probit; Standard errors clustered at household level. *p < 0.10, **p < 0.05, ***p < 0.01.

Household income is positively and significantly correlated with adoption of IGVs. This could be due to two possible reasons. First, household income could relax capital or income constraints that deter the use of improved technologies. Second, household income might serve as a buffer for farming households to deal with *ex post* production risk following adoption of IGVs, and hence encouraging them to adopt IGVs *ex ante*. However, credit access has a negative correlation with adoption of IGVs. While this result appears counterintuitive, recent evidence can help in making sense of this result. For example [33], reported that majority of farmers in Nigeria and other Sub-Saharan African countries often finance modern input purchases with cash from non-farm activities and crop sales. On the other hand, credits are often taken to complement household food availability, and credit cost servicing (transaction costs, interest and repayments) might crowd out available resources to finance the technologies and discourage investments in improved technologies [34,35].

As expected, information related variables are important determinants of adoption of IGVs. There is a positive correlation between access to extension services and adoption of IGVs, suggesting that extension access increases adoption of IGVs. As has been discussed above, the likelihood of adopting IGVs increases with residence in the tropical legumes project treatment villages and variety awareness, which are our exclusion restrictions. These results are in line with the literature on technology adoption and cement the need for strengthening information dissemination about the technologies to improve adoption of improved varieties. It is important to note that the magnitudes of the coefficients for “tropical legumes village” and “variety awareness” reported in here are slightly different from those reported in the instrument falsification tests (Table A3). This is related to the specification differences, as the aim of the selection equation is not to perfectly explain adoption, but to account for unobserved heterogeneity that could bias impacts on outcomes [36].

In terms of agronomic practices, the decision to adopt IGVs decreases with crop rotation on plots. While this is somewhat counterintuitive as groundnut is usually a crop promoted for rotation as a biological step for soil amendment, it might suggest that IGVs are less suitable for intercropping as compared to local varieties. Finally, the state dummies are interpreted relative to the reference, Bauchi state. Farmers from Kebbi and Kano states had lower likelihood of adopting IGVs, while farmers from Kastina state had a higher likelihood of adopting IGVSs compared to farmers in Bauchi state. This could reflect unobservable differences in terms of resources and ecological conditions across the states.

### 3.2 Main results

We start by discussing the relationship of IGVs adoption and household per capita groundnut consumption. The results of the full information maximum likelihood (FIML) estimation of the endogenous switching model are presented in the Appendix (Table A5 (a)). We transform per capita groundnut consumption by taking its logarithmic values to address the skewedness in its distribution. The Wald test of independent equations is not significant, confirming absence of joint dependence of the selection and outcome equations and indicating that the self-selection into the adoption of the technologies is not strong in our sample. The correlation coefficients are significant for both adopters and non-adopters. This suggests that there is endogenous switching effect. Further, these correlation coefficients have similar sign; therefore, IGV adoption had a significant impact on the corresponding outcome among adopters and non-adopters, had non-adopters chosen to adopt the technologies [25].

Table 4 presents the estimated treatment effects of adoption of IGVs on household per capita groundnut consumption. The results show that adoption of IGVs is significantly correlated with household per capita groundnut consumption. The expected log-odds of household per capita groundnut consumption for adopters is 2.03, while it is 1.99 for non-adopters. In the counterfactual scenario, adopters would have 1.91 log-odds of consuming per capita groundnut had they decided not to adopt. Hence, adoption of IGVs increases the log-odds that the household would consume groundnut by 12.75 % for adopters. The use of per capita groundnut consumption is likely to underestimate the reported percentage effect for adopters, especially if young children and toddlers account for a good proportion of household members. In this case, groundnut consumption per adult equivalent could have given precise measures of effects of adoption of IGVs, but we could not use it as the data did not include information on the ages of household members, except for the household head.

**Table 4:** Effects of IGVs on household per capita groundnut consumption: endogenous switching regressions.

Outcome	Subsamples and treatment effects	Decision stage		ATEs
		To adopt	Not to Adopt	
Household groundnut consumption (log)	Adopters of IGVs (ATT)	2.029	1.912	0.117*** (0.026)
	Non-adopters of IGVs (ATU)	1.987	1.859	0.128*** (0.015)
	Heterogeneous effects	0.042	0.053	−0.011

Notes: ATT – Average Treatment Effect on the Treated, ATU – Average Treatment Effect on the Untreated, ATE – Average Treatment Effects; Bootstrapped standard errors in parentheses; ***p < 0.01; The outcome household per capita groundnut consumption is logarithmic transformed.

In the counterfactual case, non-adopter households would have increased their household per capita groundnut consumption by about 13.66 % had they adopted the technologies. This implies that the expected effects of the technologies would have been considerable had non-adopters chosen to adopt the technologies. The magnitude of the percentage effects for current adopters and non-adopters, suggesting lack of strong heterogenous effects between the two groups. These results are consistent with previous studies on impacts of improved varieties on household welfare, for example [7], in Ethiopia and Tanzania and [8] in Nigeria.

The base heterogeneity effect for per capita groundnut consumption outcome is negative but it is small, implying that potential unobservable heterogeneity plays a minimal role in affecting the welfare of adopters. This suggests there was no hierarchal sorting that favors adopters to be above average outcomes irrespective of adoption status, but they have a higher propensity of adopting IGVs and are better off adopting the technologies than not adopting them [25,28]. This could also be the result of targeted and donated seeds of improved varieties, which is common at early trial and dissemination of new improved varieties.

The results also show differences in coefficients of the explanatory variables in the outcome equations of IGV adopters and non-adopters (Table A5). Overall, several covariates are important determinants of per capita groundnut consumption for both adopters and non-adopters, with some of the covariates having a heterogeneous association with groundnut consumption of the two groups. Among adopters of IGVs, groundnut consumption increases with the age of household head, cultivated land, livestock ownership, per capita income, and practicing crop rotation but decreases with household size. For non-adopters, per capita groundnut consumption increases with age of the household head, cultivated land and crop rotation, while decreasing with household size. Some states dummies are also significant, indicating geographic variations in groundnut consumption.

We now present results for impacts of IGV adoption on household food security, as measured by the household food insecurity access scale (HFIAS). As outlined, we estimate an endogenous switching Poisson model for the count HFIAS outcome and an endogenous switching probit for the binary food security outcome. Table A5 in the Appendix presents the FIML estimates of the two endogenous switching regressions for food security outcomes. Table 5 provides the treatment impact estimates of adoption of IGVs on these food security outcomes. Results show that adoption of IGVs reduces the levels of households’ vulnerability to food (access) insecurity by 22% points compared with the counterfactual scenario of non-adopting. Similarly, adoption of IGVs increases the probability of being food secure by about 12% points for adopters compared to the counterfactual of non-adopting.

**Table 5:** Effects of IGVs on household food security: endogenous switching Poisson and probit regressions.

Outcome	Treatment effects			
	ATT	ATU	ATE	MTE
HFIAS Score	−0.218*** (1.585)	−0.698*** (0.662)	−0.614*** (0.587)	−0.376*** (1.458)
Food secure	0.119*** (0.012)	0 .166*** (0.005)	0.155*** (0.005)	0.095*** (0.001

Notes: ATT – Average Treatment Effect on the Treated, ATU – Average Treatment Effect on the Untreated, ATE – Average Treatment Effect, and MTE – Marginal Treatment Effect; HFIAS – Household food insecurity access scale; Bootstrapped standard errors in parentheses; ***p < 0.01.

On the other hand, non-adopters would have lowered their vulnerability to the food (access) insecurity by about 70% points had they adopted IGVs. They would also have increased their likelihood of being food secure by about 17% points had they adopted IGVs. This implies that non-adopters appear to have forgone substantial benefit due to their failure to adopt the technologies, and substantial food security gains could be achieved from further promotion of the adoption of the IGVs and relaxing constraints of adoption of the technologies for non-adopters.

Finally, the results show differences in the coefficients of the explanatory variables in the outcome equations of IGV adopters and non-adopters for both HFIAS and the binary food security outcomes (Table A5). For adopters, HFIAS decreases with age and education of the household head, livestock ownership, and access to extension and credit. Similarly, food security status of adopters improves with the age of the household head, livestock ownership and extension access. Adopters who practiced crop rotation are also less likely to experience HFIAS and more likely to be food secure. On the other hand, HFIAS for non-adopters decreases with age of the household head, livestock ownership, groundnut cultivated land, household per capita income, and access to credit, but increases with the household size. Non-adopter households headed by male are less likely to experience household food insecurity access.

A potential concern with our ability to make a clear attribution is that household income may be endogenous due to reverse causality in the adoption model. High income is likely to be correlated with adoption of IGVs, while adopters of IGVs are more likely to increase their income due to the resulting high yield from cultivating IGVs. In addition, reported results might be contaminated by the so-called multicollinearity statistical problem, as (most of) the covariates are well-known and non-trivial determinants of rural income in the literature. Further, income can serve as the mediation between adoption of IGVs and food security. To attenuate these concerns, we exclude income level from our covariates and examine if our results are robust to omission of per capita income. The new results of the full information maximum likelihood (FIML) from the endogenous switching regression are presented in the Appendix (Table A6). The corresponding results for the effects of IGVs on household per capita groundnut consumption and food security outcomes are presented in Table 6, Table 7, respectively. Results show that adoption of IGVs increases the log-odds that the household would consume groundnut by 8.44 % (Table 6). Similarly, adoption of IGVs decreases households’ vulnerability to food (access) insecurity by about 54% points, while increasing the likelihood of adopters being food secure by about 14% points compared to the counterfactual of non-adopting IGVs (Table 7). Altogether, the main results remain robust for the exclusion of income from the covariates.

**Table 6:** Effects of IGVs on household per capita groundnut consumption: endogenous switching regression (household income excluded from regression).

Outcome	Subsamples and treatment effects	Decision stage		ATEs
		To adopt	Not to Adopt	
Per capita groundnut consumption	Adopters of IGVs (ATT)	1.993	1.912	0.081*** (0.023)
	Non-adopters of IGVs (ATU)	1.955	1.899	0.056*** (0.015)
	Heterogeneous effects	0.038	0.013	0.025

Notes: ATT – Average Treatment Effect on the Treated, ATU – Average Treatment Effect on the Untreated, ATE – Average Treatment Effects; Bootstrapped standard errors in parentheses; ***p < 0.01; The outcome household per capita groundnut consumption is logarithmic transformed.

**Table 7:** Effects of IGVs on household food security: endogenous switching poisson and probit regressions (household income excluded from regression).

Outcome	Treatment effects			
	ATT	ATU	ATE	MTE
HFIAS Score	−0.543*** (1.172)	−0.089*** (0.550)	−0.818*** (0.491)	−0.363*** (1.223)
Food secure	0.139*** (0.217)	0 .156*** (0.213)	0.153*** (0.212)	0.082*** (0.015)

Notes: ATT – Average Treatment Effect on the Treated, ATU – Average Treatment Effect on the Untreated, ATE – Average Treatment Effect, and MTE – Marginal Treatment Effect; HFIAS – Household food insecurity access scale; Bootstrapped standard errors in parentheses; ***p < 0.01.

Why may adoption of IGVs improve household food security? As outlined, groundnut can contribute to household food and nutrition security directly and indirectly. Directly, groundnut is an important source of affordable protein for the rural and urban poor whose diets often rely on starchy grains, roots and tubers. As such, consumption of groundnut supplies protein, healthy fats, vitamins and micronutrients to rural people [13]. Supporting this line of contribution, IGVs can improve yield and increase food availability for household consumption. Evidently, the average groundnut yield per hectare for adopters of IGVs (1312 kg/ha) is significantly higher than that for non-adopters (1129 kg/ha) (independent two-sample *t*-test; *p* < 0.001). Indirectly, groundnut is an important cash crop and can help households to gain more income that increases their purchasing power to diversify diets through market purchase. Our data show that adopters of IGVs (74 %) are more likely to participate in markets to sell groundnut than non-adopters of the technologies (62 %) (independent two-sample *t*-test; *p* < 0.001). Net returns from groundnut production valued at local producer prices in naira (₦) are significantly higher for adopters of IGVs (₦341,000) than for non-adopters (₦117,000) (independent two-sample *t*-test; *p* < 0.05). Further, groundnut production improves soil fertility in drylands of sub-Saharan Africa (SSA) due to its high potential to fix nitrogen, potentially contributing to the productivity and health of the overall farm system of households.

## 4 Conclusion and discussion

Adoption of improved agricultural technologies has long been recognized as a critical pathway for reducing rural poverty and improving household welfare in developing countries. Using detailed household and plot level data from over 1300 households in Nigeria, this study has investigated the correlation between adoption of improved groundnut varieties (IGVs) and household per capita groundnut consumption and food security outcomes. Food security outcomes are measured using the household food insecurity access scale (HFIAS) and a binary food security outcome, whether a household experienced none of the food insecurity conditions. We use endogenous switching regression models to control for unobserved endogeneity and selection biases.

We find that adoption of IGVs is significantly correlated with higher household per capita groundnut consumption and improved food security outcomes, which are critical indicators of the pathway to the achievement of the goals of ‘no poverty’ and ‘zero hunger’. Results show that about 30 % of groundnut plots are planted with improved varieties, and the adoption of IGVs increases the likelihood that the household would consume groundnut by about 13 % and reduces the probability of households' vulnerability to food (access) insecurity by 22 %. Generally, reported results provide evidence of a consistent and strong positive relationship between adoption of IGVs and considered outcome variables. They also survive sensitivity analyses with respect to income and its potential multicollinearity with other explanatory variables. Nevertheless, we acknowledge that establishing a neat causal attribution remains challenging based on cross-sectional data. We seek to attenuate concerns about unobserved heterogeneity using the endogenous switching regression models, but the imperfect nature of our instruments means that we cannot rule out all potential concerns about endogeneity. Yet, we believe that reported results are still informative to understand the interplay between adoption of IGVs and their welfare consequences, even in the absence of clear causality, in Nigeria.

With this consideration, our results have important implications for efforts aiming at promoting adoption of IGVs. Results from the counterfactual analysis indicate non-adopting households would have enjoyed comparable benefits in terms of per capita groundnut consumption and food security outcomes, had they adopted the technologies. This result is particularly important to inform efforts to promote adoption of IGVs, such as scaling of extension services, for present non-adopters in Nigeria. To further support such efforts, our study provides evidence on important determinants of adoption of IGVs that can be used to guide identification of barriers and opportunities for improving adoption of IGVs. Importantly, the positive and significant results of information related factors and farmer awareness of improved varieties on their adoption suggest that increasing access to information on the technologies need to be a key part of any effort targeting at nudging the adoption and diffusion of IGVs in Nigeria. Overall, considerable investments need to be made to improve information dissemination and technology scaling mechanisms to reach smallholder farmers in remote rural areas, while developing groundnut seed systems to ensure the readily availability of quality improved seeds at affordable prices.

## Declaration of competing interest

The authors declare no conflict of interest with or involvement in any organization or entity with financial, scientific, or otherwise in the subject matter or materials discussed in this manuscript.

## Figures and Tables

**Table A1:** Adoption levels of popular groundnut varieties

Groundnut variety	Variety category	Year of release	Adoption rate (% households)
**SAMNUT-10**	Improved	1988	0.5
**SAMNUT-11**	Improved	1988	0.2
**SAMNUT-21**	Improved	2001	1.3
**SAMNUT-22**	Improved	2001	0.06
**SAMNUT-23**	Improved	2001	0.83
**SAMNUT-24**	Improved	2011	25.62
**SAMNUT-25**	Improved	2013	0.47
**SAMNUT-26**	Improved	2013	1.71
**Maiborgo**	Local	n/a	9.67
**Yardakar**	Local	n/a	9.50
**Kampala**	Local	n/a	2.77
**Kwankwaso**	Local	n/a	12.63
**Burguwa**	Local	n/a	3.07

**Table A2:** Household Food Insecurity Access Scale (HFIAS) measurement tool

1.During the last four weeks, did you worry that your household would not have enough food? 0 = No (skip to Q2) 1 = Yes. If yes, how often did this happen? Codes: 1 = Rarely (once or twice in the past four weeks), 2 = Sometimes (three to ten times in the past four weeks), 3 = Often (more than ten times in the past four weeks)
2. During the last four weeks, were you or any household member not able to eat the kinds of foods you preferred because of a lack of resources? 0 = No (skip to Q3) 1 = Yes. If yes, how often did this happen? Codes: 1 = Rarely (once or twice in the past four weeks), 2 = Sometimes (three to ten times in the past four weeks), 3 = Often (more than ten times in the past four weeks)
3. During the last four weeks, did you or any household member have to eat a limited variety of foods due to lack of resources to obtain other types of foods? 0 = No (skip to Q4) 1 = Yes. If yes, how often did this happen? Codes: 1 = Rarely (once or twice in the past four weeks), 2 = Sometimes (three to ten times in the past four weeks), 3 = Often (more than ten times in the past four weeks)
4. During the last four weeks, did you or any household member eat food that you preferred not to eat because of a lack of resources to obtain other types of food? 0 = No (skip to Q5) 1 = Yes. If yes, how often did this happen? Codes: 1 = Rarely (once or twice in the past four weeks), 2 = Sometimes (three to ten times in the past four weeks), 3 = Often (more than ten times in the past four weeks)
5. During the last four weeks, did you or any household member eat a smaller meal portion than you felt you needed because there was not enough food? 0 = No (skip to Q6) 1 = Yes. If yes, how often did this happen? Codes: 1 = Rarely (once or twice in the past four weeks), 2 = Sometimes (three to ten times in the past four weeks), 3 = Often (more than ten times in the past four weeks)
6. During the last four weeks, did you or any other household member eat fewer number of meals in a day because there was not enough food? 0 = No (skip to Q7) 1 = Yes. If yes, how often did this happen? Codes: 1 = Rarely (once or twice in the past four weeks), 2 = Sometimes (three to ten times in the past four weeks), 3 = Often (more than ten times in the past four weeks)
7. During the last four weeks, was there ever no food at all in your household because there were no resources to get more food? 0 = No (skip to Q8) 1 = Yes, If yes, how often did this happen? Codes: 1 = Rarely (once or twice in the past four weeks), 2 = Sometimes (three to ten times in the past four weeks), 3 = Often (more than ten times in the past four weeks)
8. During the last four weeks, did you or any household member go to sleep at night hungry because there was not enough food? 0 = No (skip to Q9) 1 = Yes. If yes, how often did this happen? Codes: 1 = Rarely (once or twice in the past four weeks), 2 = Sometimes (three to ten times in the past four weeks), 3 = Often (more than ten times in the past four weeks)
9. During the last four weeks, did you or any household member go a whole day without eating anything because there was not enough food? 0 = No 1 = Yes, If yes, how often did this happen? Codes: 1 = Rarely (once or twice in the past four weeks), 2 = Sometimes (three to ten times in the past four weeks), 3 = Often (more than ten times in the past four weeks)

**Table A3:** Instrument falsification tests on the validity of the selection instrument**s** (parameter estimates)

Variables	IGV (Probit)	Per capita groundnut consumption (OLS)	HFIAS score (Poisson)	Food secure (Probit)
***Instruments***				
Tropical legumes village	0.436*** (0.129)	0.14 (0.142)	−0.143 (0.157)	−0.097 (0.112)
Variety aware	1.623*** (0.093)	−0.197 (0.177)	−0.161 (0.177)	−0.023 (0.127)
*Controls*	Yes	Yes	Yes	Yes
Constant	−0.634 (0.677)	2.545 (0.764)	3.403*** (0.899)	−1.125* (0.672)
χ^2^	450.30***		130.853***	35.937***
F		13.501		
Observations	1694	1186	1186	1186

Notes: The outcomes (Groundnut consumption, HFIAS score and Food secure) are for non-adopters. All control variables are included in the models, but parameters are not reported to save space. Standard errors clustered at household level in parentheses. IGV – Improved groundnut variety; HFIAS – Household food insecurity access scale. *p < 0.1, **p < 0.05, ***p < 0.01.

**Table A4:** Additional statistical tests for the appropriateness of the IV approach and instruments

Statistical test	Per capita groundnut consumption	HFIAS score	Food secure
***Test of exogeneity***			
Wu-Hausman F statistic p-value	6.730 0.010	5.514 0.019	0.016 0.899
Durbin-Wu-Hausman χ^2^ p-value	6.783 0.009	5.551 0.018	0.016 0.898
***Weak Identification tests***			
Kleibergen-Paap rk Wald F statistic p-value	294.29> gmin^a^ 19.93 0.000	137.819> gmin^a^ 19.93 0.000	137.819> gmin^a^ 19.93 0.000
***Over-identification test***			
Anderson-Rubin Wald test χ^2^ p-value	6.940 0.031	16.13 0.000	2.78 0.249
Hansen's J statistic χ^2^ p-value	4.312 0.037	8.163 0.004	2.117 0.146

HFIAS – Household food insecurity access scale. ^a^Based on Stock and Yogo (2005), critical value = 19.93 *K*_*2*_ = 2; *n* = 1; *r* = 0.10 at 5 % significance level.

**Table A5:** Full information maximum likelihood estimates of the endogenous switching regression models

	(a) Per capita groundnut consumption		(b) Household food insecurity access scale		(c) Food secure	
	Non-adopters	Adopters	Non-adopters	Adopters	Non-adopters	Adopters
Age of head	0.323* (0.209)	0.442* (0.259)	−1.285*** (0.160)	−1.005** (0.397)	0.395** (0.188)	0.540* (0.311)
Male household head	0.048 (0.328)	−0.025 (0.221)	−1.105*** (0.137)	0.044 (0.163)	0.102 (0.214)	0.074 (0.221)
Education of head	−0.108 (0.170)	−0.098 (0.158)	0.13 (0.133)	−0.335*** (0.111)	−0.161 (0.132)	0.003 (0.197)
Household size	−1.111*** (0.095)	−0.932*** (0.102)	0.445*** (0.075)	0.046 (0.209)	−0.117 (0.087)	−0.156 (0.135)
Livestock TLU	0.007 (0.072)	0.120* (0.079)	−0.140** (0.072)	−0.269*** (0.083)	0.031 (0.067)	0.212** (0.100)
Cultivated land (ha)	0.035* (0.029)	0.018** (0.017)	−0.086*** (0.004)	−0.019 (0.040)	0.004 (0.027)	0.041 (0.026)
Extension access	−0.031 (0.143)	−0.203 (0.169)	0.075 (0.117)	−1.401*** (0.215)	0.180* (0.107)	0.867*** (0.236)
Crop rotation	0.351** (0.143)	0.200** (0.128)	−0.011 (0.094)	−0.686*** (0.146)	0.006 (0.097)	0.392** (0.168)
Log household per capita income	0.001 (0.0002)	0.001** (0.0002)	−0.001*** (0.0004)	0.00005 (0.0001)	0.001 (0.0004)	−0.0001 (0.0002)
Log credit amount	−0.099 (0.063)	−0.102 (0.088)	−0.137*** (0.022)	−0.190*** (0.053)	−0.038 (0.054)	−0.112 (0.078)
**State dummies**						
Kebbi	0.205 (0.285)	−0.288 (0.264)	−0.196 (0.125)	2.342*** (0.302)	0.16 (0.170)	−1.148*** (0.344)
Kastina	0.531** (0.240)	−0.531*** (0.204)	−0.647*** (0.178)	0.604** (0.243)	0.181 (0.180)	−0.749** (0.293)
Kano	0.297 (0.249)	0.264 (0.240)	0.579*** (0.143)	−0.22 (0.722)	−0.223 (0.176)	−0.362 (0.367)
Jigwa	0.168 (0.271)	−0.587** (0.244)Para Run-on-->	0.266** (0.105)	1.323*** (0.280)	−0.223 (0.179)	−1.280*** (0.299)
Constant	2.674*** (0.741)	2.368** (0.992)	4.311*** (0.561)	0.679 (1.030)	−1.189* (0.693)	0.006 (1.118)
Model significance Wald χ2 p-value	211.66 0.000				348.39 0.000	
**Model diagnosis**						
Lnsigma (0/1)	0.234*** (0.041)	0.008** (0.076)	0.627*** (0.040)	0.611*** (0.075)		
	
Rho	0.090 (0.154)	0.140 (0.136)	−0.055 (0.147)	−0.028 (0.107)		
	
Ortho					−0.157 (0.209)	−0.07 (0.199)
	
Test of independent equation Wald χ2 p-value	1.32 0.517				0.68 0.713	
Observations	1186	508	1186	508	1186	508

Standard errors clustered at household level in parentheses; *p < 0.1, **p < 0.05, ***p < 0.01.

**Table A6:** Full information maximum likelihood estimates of the endogenous switching regressions (income excluded from regressors)

	Per capita groundnut consumption		Household food insecurity access scale		Food secure	
	Non-adopters	Adopters	Non-adopters	Adopters	Non-adopters	Adopters
Age of head	0.335* (0.208)	0.457* (0.258)	−0.232 (0.153)	−0.955*** (0.345)	0.403** (0.186)	0.525* (0.310)
Male household head	0.045 (0.328)	−0.041 (0.221)	−0.712*** (0.099)	0.012 (0.154)	0.114 (0.213)	0.084 (0.224)
Education of head	−0.088 (0.170)	−0.089 (0.155)	0.053 (0.066)	−0.334*** (0.117)	−0.144 (0.131)	−0.003 (0.194)
Household size	−1.114*** (0.095)	−0.938*** (0.101)	0.099** (0.049)	0.019 (0.188)	−0.129 (0.086)	−0.15 (0.135)
Livestock TLU	0.039 (0.071)	0.127** (0.079)	0.096 (0.107)	−0.269*** (0.085)	0.06 (0.064)	0.214** (0.100)
Cultivated land (ha)	0.032* (0.029)	0.017 (0.017)	−0.076*** (0.005)	−0.021 (0.038)	0.004 (0.027)	0.04 (0.026)
Extension access	−0.045 (0.143)	−0.186 (0.168)	−0.223*** (0.080)	−1.408*** (0.215)	0.187* (0.107)	0.859*** (0.236)
Crop rotation	0.338** (0.144)	0.198** (0.126)	−0.04 (0.074)	−0.668*** (0.140)	0.026 (0.097)	0.379** (0.165)
Log credit amount	−0.103 (0.063)	−0.103 (0.088)	0.037 (0.035)	−0.194*** (0.055)	−0.036 (0.054)	−0.113 (0.077)
**State dummies**						
Kebbi	0.218 (0.285)	−0.289 (0.265)	−0.184 (0.128)	2.311*** (0.268)	0.148 (0.170)	−1.145*** (0.344)
Kastina	0.625*** (0.241)	−0.504** (0.204)	−0.920*** (0.173)	0.587** (0.234)	0.212 (0.179)	−0.747** (0.291)
Kano	0.335 (0.250)	0.268 (0.242)	−0.198* (0.116)	−0.221 (0.887)	−0.216 (0.176)	−0.362 (0.367)
Jigwa	0.208 (0.272)	−0.577** (0.245)	−0.329** (0.129)	1.284*** (0.244)	−0.224 (0.179)	−1.269*** (0.300)
Constant	2.612*** (0.738)	2.294** (0.992)	0.938 (0.572)	0.597 (0.992)	−1.203* (0.688)	0.011 (1.114)
Model significance Wald χ2 p-value	192.05 0.000				343.30 0.000	
Lnsigma (0/1)	0.240*** (0.041)	0.010 (0.076)	0.647*** (0.041)	0.608*** (0.075)		
	
Rho	0.088 (0.154)	0.167 (0.134)	−0.062 (0.136)	−0.032 (0.112)		
	
Ortho					−0.175 (0.216)	−0.047 (0.198)
			
Test of independent equation Wald χ2 p-value	1.78 0.411				0.70 0.705	
Observations	1186	508	1186	508	1186	508

Standard errors clustered at household level in parentheses; *p < 0.1, **p < 0.05, ***p < 0.01.

## Data Availability

Data will be made available on request.

## Footnotes

1 Missing households do not statistically differ from study households across relevant socioeconomic characteristics.
